# PARP Inhibitors for Sensitization of Alkylation Chemotherapy in Glioblastoma: Impact of Blood-Brain Barrier and Molecular Heterogeneity

**DOI:** 10.3389/fonc.2018.00670

**Published:** 2019-01-22

**Authors:** Shiv K. Gupta, Emily J. Smith, Ann C. Mladek, Shulan Tian, Paul A. Decker, Sani H. Kizilbash, Gaspar J. Kitange, Jann N. Sarkaria

**Affiliations:** ^1^Departments of Radiation Oncology, Mayo Clinic, Rochester, MN, United States; ^2^Division of Biomedical Statistics and Informatics, Department of Health Sciences Research, Mayo Clinic, Rochester, MN, United States; ^3^Departments of Oncology, Mayo Clinic, Rochester, MN, United States

**Keywords:** PARP (poly(ADP-ribose) polymerase, chemo-radiation sensitivity, DNA Damage, replication stress, DNA repair activity

## Abstract

Prognosis of patients with glioblastoma (GBM) remains dismal despite maximal surgical resection followed by aggressive chemo-radiation therapy. Almost every GBM, regardless of genotype, relapses as aggressive recurrent disease. Sensitization of GBM cells to chemo-radiation is expected to extend survival of patients with GBM by enhancing treatment efficacy. The PARP family of enzymes has a pleiotropic role in DNA repair and metabolism and has emerged as an attractive target for sensitization of cancer cells to genotoxic therapies. However, despite promising results from a number of preclinical studies, progress of clinical trials involving PARP inhibitors (PARPI) has been slower in GBM as compared to other malignancies. Preclinical *in vivo* studies have uncovered limitations of PARPI-mediated targeting of base excision repair, considered to be the likely mechanism of sensitization for temozolomide (TMZ)-resistant GBM. Nevertheless, PARPI remain a promising sensitizing approach for at least a subset of GBM tumors that are inherently sensitive to TMZ. Our PDX preclinical trial has helped delineate *MGMT* promoter hyper-methylation as a biomarker of the PARPI veliparib-mediated sensitization. In clinical trials, *MGMT* promoter hyper-methylation now is being studied as a potential predictive biomarker not only for response to TMZ therapy alone, but also PARPI-mediated sensitization of TMZ therapy. Besides the combination approach being investigated, IDH1/2 mutant gliomas associated with 2-hydroxygluterate (2HG)-mediated homologous recombination (HR) defect may potentially benefit from PARPI monotherapy. In this article, we discuss existing results and provide additional data in support of potential alternative mechanisms of sensitization that would help identify potential biomarkers for PARPI-based therapeutic approaches to GBM.

## Background

Glioblastoma (GBM) is a fatal disease with less than 2% of patients surviving 5 years after initial diagnosis and treatment (1, 2). GBM therapy, which includes aggressive surgical resection, high dose external beam radiation therapy (RT) and temozolomide (TMZ) chemotherapy, is associated with a median time to progression of approximately 6 months and a median overall survival of 15 months (3). Sensitizing strategies to enhance efficacy of radiation and chemotherapy may prolong patient survival. TMZ, used as standard of care for newly diagnosed GBM, is a mono-alkylating agent that induces cytotoxic lesions including N_7_-methylguanine (N7MeG), N_3_-methyladenine (N3MeA) and O_6_-methylguanine (O6MeG) (4, 5). N7MeG and N3MeA are repaired by base-excision repair (BER) and contribute minimally to overall cytotoxicity of TMZ, while O6MeG is repaired by O_6_-methylguanine-DNA-methyl transferase (MGMT), found suppressed by promoter methylation in ~40% of GBM tumors. Lack of MGMT expression results in persistent O6MeG lesions that trigger replicative stress and cytotoxicity via futile cycles of mismatch repair (MMR) (5, 6). The poly (ADP ribose) polymerase (PARP) family of enzymes coordinates the DNA damage response. Binding of PARP1 to nicked DNA provides the necessary scaffold that recruits BER components (7, 8). Therefore, PARP inhibitors (PARPI) were thought to potentiate TMZ by disrupting BER (9). Indeed, PARPI potentiate TMZ efficacy in numerous pre-clinical models (4, 9), providing a rationale for clinical development of PARPI to potentiate TMZ therapy in GBM. In addition to the established role of PARP in BER, destabilization of stalled replication forks by allosteric trapping of PARP also contributes toward mechanisms of TMZ sensitization by PARPI (10).

However, like other novel drugs for GBM, several promising PARPI agents have limited distribution across the blood-brain barrier (BBB) or demonstrate heterogeneous *in vivo* response (11). For example, talazoparib and rucaparib are potent PARPI that are substrates for the efflux transporters P-glycoprotein (PgP) and/or breast cancer resistance protein (BCRP) that are active in brain endothelial cells (12, 13). In keeping with poor brain penetration, these drugs have limited distribution and no appreciable TMZ sensitization in orthotopically implanted GBM patient-derived xenografts (PDXs). In contrast, the PARPI veliparib is brain penetrant and an effective TMZ-sensitizer in a subset of GBM PDX models (4, 14, 15). Based on previously published data and additional experimental results, the focus of this article is to explore potential biomarkers critical to a PARPI-based sensitization approach to GBM therapy.

### Discordance Between *in vitro* Versus *in vivo* Preclinical Data

Numerous preclinical studies have investigated the combination of PARPI with RT, TMZ or RT/TMZ and other chemotherapy agents in glioma models (14, 16, 17). Models including established glioma cell lines (16, 18–20), zebrafish embryos (21), genetically engineered mouse models (GEMM) (22) and PDXs (14) have been used. While each of these models has helped to characterize PARPI combinations, discordance between *in vitro* vs. *in vivo* data needs to be considered when developing therapies based on preclinical studies. Specifically, the *in vitro* sensitizing effects of the PARPI veliparib were pronounced in TMZ-resistant models, while these models did not benefit from the combination *in vivo*. In contrast, *in vivo* sensitization by veliparib was pronounced in TMZ-sensitive models, although the *in vitro* sensitization was limited (4). This discordance is due to *in vivo* drug achievability, which was lower than concentrations required for DNA damage induction in resistant tumors (4). These results highlight the importance of using clinically relevant concentrations of both TMZ and PARPI for *in vitro* assays and raise the possibility that molecular mechanisms defined by using supratherapeutic drug concentrations may not be applicable to *in vivo* sensitization.

PDX models are translationally relevant because they preserve the genetic characteristics of the tumor, and orthotopically implanted PDXs represent tumor microenvironment and vascular structures found in human GBM (23–25). Furthermore, pharmacokinetic profiles of PARPI in murine models mimic drug exposures reported in human clinical trials (12, 18). GEMMs are ideal to study gliomagenesis; however, GEMMs cannot recapitulate genetic heterogeneity or epigenetic features, such as *MGMT* promoter methylation found in human GBM. Use of large panels of PDXs for drug evaluation may accurately model tumor heterogeneity and the variability in response. As reported previously, veliparib-mediated *in vivo* sensitization is associated with inherent TMZ sensitivity (4, 14). This concept was further tested in a preclinical PDX trial using orthotopic therapy models of 28 different GBM PDX lines with or without *MGMT* promoter methylation, a marker of TMZ sensitivity (15). In this study, profound survival extension with TMZ/veliparib over TMZ alone was observed in ~45% of PDX models with *MGMT* hyper-methylation, while *MGMT* unmethylated models had no meaningful survival benefit (15). This result helped delineate *MGMT* promoter methylation as a predictive biomarker for veliparib-mediated sensitization (15).

### Mechanism of PARPI-Mediated Sensitization:

Understanding mechanisms of sensitization is important to delineate biomarkers and new therapeutic targets. Synthetic lethality of PARPI with HR is the hallmark of single-agent PARPI therapy in breast and ovarian cancers (26, 27). PARPI also potentiate efficacy of genotoxic agents, including DNA alkylating agents and RT (28). Mechanistically, enzymatic activation of PARP consumes NAD+ and generates poly-ADP-ribose (PAR) moieties to modify interacting proteins and itself via a phenomenon known as PARylation (29). PARP auto-PARylation at DNA lesions initiates recruitment of repair proteins, while also keeping PARP-DNA interactions unstable allowing repair machinery access to the lesion (7, 30). PARPI blocks auto-PARylation and prevents dissociation of PARP-DNA interactions, thereby trapping PARP at the damage site, leading to replicative stress and replication-associated double strand DNA breaks (10, 31).

Prior studies suggest that PARPI-mediated *in vivo* sensitization of TMZ depends on replicative stress caused by persistent O6MeG (4, 14, 15). The significance of PARP trapping to O6MeG-mediated replicative stress is unclear as PARP is known to engage at N7MeG and N3MeA lesions. PARPI-mediated BER inhibition and PARP trapping contribute more robustly at supratherapeutic drug concentrations used *in vitro*; whether PARPI concentrations achievable *in vivo* induce detectable PARP trapping remains to be seen (30, 32, 33). Furthermore, PARPI with high trapping capacity are not well tolerated in combination with TMZ (30), and dose-reduced regimens tested have not shown greater sensitization than veliparib, a weak trapping agent (12–15). In a head-to-head comparison of PARPI agents, the trapping capacity was found to be inversely correlated with *in vivo* efficacy (30), suggesting that the trapping ability of PARPI may not be fully exploited for TMZ sensitization. However, this can be important to PARPI monotherapy or combinations where higher doses of trapping agents can be safely administered. Robust *in vitro* radio-sensitizing effects of PARPI talazoparib used at clinically relevant concentrations have been reported (34). However, evaluation of radio-sensitizing effects of talazoparib in *in vivo* orthotopic GBM models will be important as talazoparib concentrations in intracranial tumors may not reach clinically relevant concentrations based on plasma level (12).

**Figure 1:**
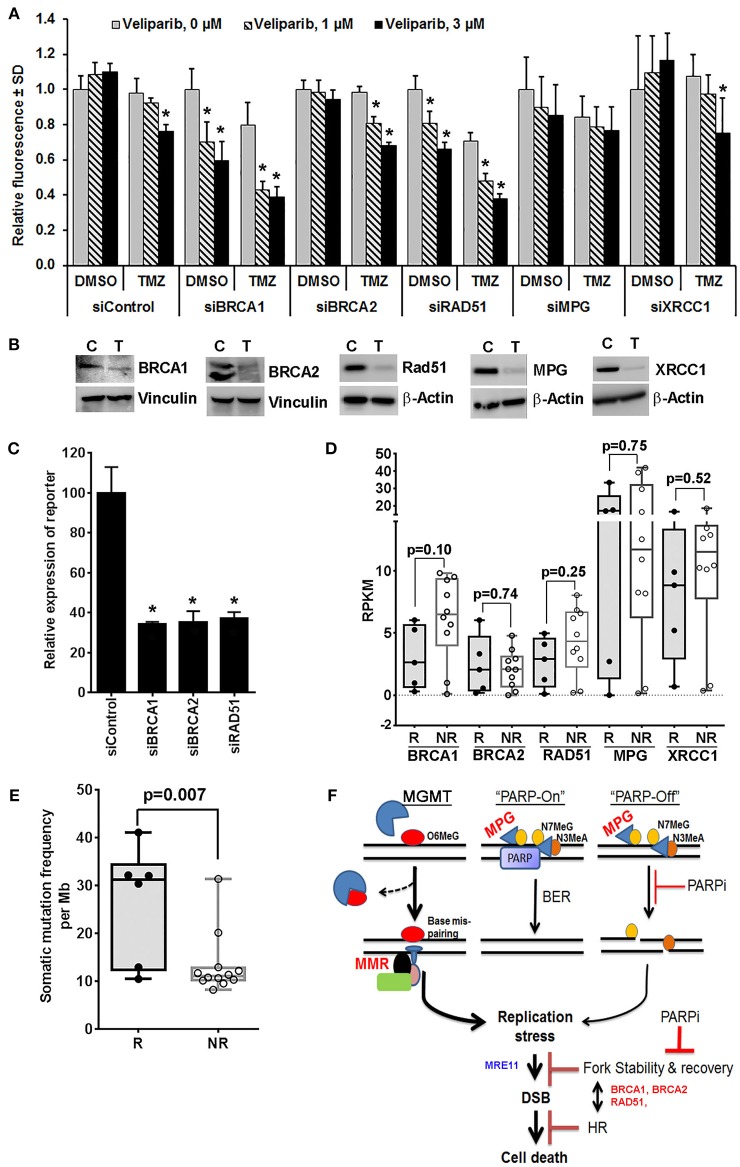
Mechanistic insights into veliparib-mediated sensitization of TMZ therapy in GBM cells (see Supplementary Material for Materials and Methods). **(A)** Effect of homologous recombination (HR) vs. base excision repair (BER) pathway disruption on veliparib-mediated sensitization in U251TMZ cells. Cells transfected with specified siRNA were seeded in 96 well plates (500 cells per well), treated with the vehicle or 30 μM TMZ ± 1 or 3 μM veliparib for 5 days and cell growth measured by CyQuant assay. Bar graphs demonstrate change in average fluorescence intensity relative to control, error bars represent standard deviation calculated from 3 replicates in a representative experiment, and *p < 0.05 compared to corresponding control. **(B)** Western blot analysis to determine level of knockdown for cells used in **(A)**, lanes marked with T represent cells transfected with targeted siRNA and C represent cells transfected with control siRNA. **(C)** Bar graphs showing effects of BRCA1, BRCA2 or RAD51 knockdown on HR efficiency. U251TMZ-DRGFP cells were transfected with specific siRNA along with plasmid pCBASceI encoding I-SceI restriction enzyme and incubated for 72 h followed by quantification of GFP expressing population by FACS analysis. *p < 0.05 as compared to control. **(D)** Box plots showing expression levels for specified genes, RKPM values were extrapolated from RNA-Seq data of PDX lines differentially sensitized by veliparib in preclinical PDX trial. Data shown are for 5 of 6 responders (R) lines, which had significantly improved survival vs. 10 of 16 non-responder (NR) lines, which had no significant survival improvement with veliparib/TMZ therapy over TMZ alone in preclinical PDX trial reported previously. two tailed *p*-values reported were calculated by unpaired *t*-test. **(E)** Box plots showing mutation burden based on whole exome seq data available for 21 MGMT methylated PDX lines used in PDX pre-clinical trial and plotted grouped as TMZ/veliparib Responsive (R) vs. Non-responsive (NR) models. SNVs and INDELs across 346 genes involved in DNA damage recognition or repair were analyzed for mutation burden, two tailed *p*-values reported were calculated by unpaired *t*-test. **(F)** Hypothetical model of potential mechanism of the sensitizing effect of PARP inhibition on TMZ therapy *in vivo*. O6MeG, O_6_-methylguanine; N7MeG, N_7_-methylguanine; N3MeA, N_3_-methyladenine; MPG, methyl purine glycosylase; PARP, poly-ADP-ribose polymerase; PARPi, poly-ADP-ribose polymerase inhibitor; MMR, mismatch repair; DSB, double strand DNA breaks; MRE11, Meiotic Recombination 11 Homolog; BRCA1 and BRCA2, BReast CAncer genes 1 and 2; and HR, homologous recombination.

Veliparib-mediated *in vivo* sensitization is limited to a subset of tumors that are inherently sensitive to TMZ (4, 14), suggesting that N7MeG or N3MeA lesions may have little effect on *in vivo* sensitization (4, 12). Consistent with this idea, here we demonstrate that depletion of XRCC1 or MPG, the essential proteins in the BER pathway, had no further increase in sensitization at clinically relevant veliparib concentrations in U251TMZ cells (Figures 1A,B). However, knockdown (KD) of *BRCA1* or *RAD51* in U251TMZ cells increased sensitivity to veliparib or TMZ alone, but also led to robust TMZ sensitization (Figures 1A,B). Surprisingly, *BRCA2* KD had no increase in sensitivity toward veliparib or TMZ; additionally, veliparib-mediated sensitization was modest in *BRCA2* KD cells compared to that in *BRCA1* or *RAD51* KD cells (Figures 1A,B). The differential response among *BRCA2* vs. *BRCA1* or *RAD51* KD cells was intriguing as HR efficiency was equally suppressed in *BRCA1, BRCA2* or *RAD51* KD cells (Figure 1C). BRCA1, BRCA2 and RAD51 have also been reported to regulate replication fork stability, a function considered unrelated to HR (35, 36). Thus, compromised fork protection by BRCA1 or RAD51 depletion can be a new mechanism of PARPI-mediated TMZ sensitization.

Available RNA-Seq data from the PDX lines, used in the previously reported preclinical trial (15), showed that among analyzed HR and BER pathway genes, the expression of *BRCA1* was trending lower in all five TMZ/veliparib responsive lines as compared to 10 non-responsive GBM lines that were analyzed (*p* = 0.10, Figure 1D). Interestingly, the *BRCA1* expression, found upregulated in GBM, appears to be a prognostic factor in a Rembrandt GBM patient data set (Supplementary Figure 1). Our limited RNA Seq results suggest that low *BRCA1* expression could be useful to identify tumors likely to respond to PARPI-mediated sensitization. The mechanism of BRCA1 downregulation in responder PDXs is not yet clear, although the promoter hyper-methylation, microRNA or the epigenetic modifier RBBP4, have been previously reported to influence *BRCA1* expression (37–40). Similarly, analysis of available whole exome-seq data for PDX lines used in our preclinical trial showed a significantly higher average mutation burden in responder lines than non-responder lines (Figure 1E), suggesting that the GBM tumors with genomic instability are likely to respond to PARPI-mediated sensitization. This idea that *BRCA1* downregulation in responder PDXs correlates with increased mutation frequency will need further validation.

TMZ induces replicative stress via futile attempts of MMR at O6MeG:T mismatches, while PARPI may further enhance the stress by compromising stability of stalled replication forks (41, 42). Association of BRCA1 levels with TMZ/veliparib response in the PDX trial indicates that BRCA1 synthetic lethality with PARPI can be important to fork protection (43) in the context of TMZ/veliparib treatment. Understanding the relationship between PARP and other proteins involved in fork protection may reveal key determinants of PARPI-mediated sensitization. Figure 1F shows an overview of potential mechanisms of PARPI-mediated sensitization.

### Efflux Liability and Delivery Across BBB: A key Determinant of *in vivo* Sensitizing Effects

Drug exclusion from the brain by the BBB undermines the efficacy of many CNS-directed pharmaceutical agents including PARPI (11, 44). The BBB is a complex neurovascular unit comprised of specialized brain capillary endothelium expressing ATP-binding cassette transporters. The distribution of contrast enhancement agents on magnetic resonance imaging (MRI) is commonly used to assess BBB integrity in gliomas. However, infiltrating GBM cells invade brain tissues beyond margins of contrast enhancement (45, 46). The invasive front of GBM tumors is not accessible to cyto-reductive surgery or chemotherapies that do not adequately penetrate the brain. BBB breakdown in GBM is regional and heterogeneous (44), and therefore drug distribution can be significantly lower at infiltrating edges as compared to the necrotic tumor core (44). Thus, delivery to infiltrating glioma cells is limited for many chemotherapy drugs in GBM (13, 47–49). Considerable effort has been made to understand the brain pharmacokinetics of PARPI, and several PARPI, especially the trapping agents, talazoparib and rucaparib, have efflux liabilities at the BBB and therefore lack sensitizing activity in orthotopic tumor models despite their excellent activity in heterotopic tumor models (13, 30). These findings are consistent with the notion that the delivery of targeted drugs into normal brain or orthotopically implanted tumors can model their efficacy in GBM (11, 49).

We have previously reported that the talazoparib concentration in a normal mouse brain (0.5 ng/g, or 1.3 nmol/L) after drug administration was lower than required for effective PARP inhibition *in vitro*. Comparing the pharmacokinetics of talazoparib to other PARPI in healthy rodents, the brain-to-plasma concentration ratio for talazoparib (0.02) was lower than that of rucaparib (0.11), which also lacks efficacy in orthotopic glioma models (13). Olaparib is another PARP trapping agent known to have efflux liability and restricted delivery across the BBB (50, 51). Although a phase I clinical trial in patients with recurrent GBM has shown that olaparib can reach the core and the margins of GBM tumors (50), this data has to be interpreted cautiously because GBM cells invade tissues beyond the margins defined by the MRI. Veliparib, on the other hand, has a much higher brain-to-plasma concentration ratio (0.47) than either talazoparib or rucaparib despite the efflux liability of veliparib to MDR1 and BCRP (15, 52). Furthermore, unlike talazoparib and rucaparib, veliparib sensitized orthotopic GBM models despite being significantly less potent in terms of PARP trapping (15). A comparison of the properties of drugs from the same class provides insight on the relative significance of variables such as drug potency, BBB penetrability, and efflux liability for efficacy in orthotopic glioma models. These considerations emphasize the importance of brain pharmacokinetics, drug tolerability, and efficacy evaluation in animal models for the successful design of novel therapies for GBM.

### Clinical Trials of PARPI in GBM

PARPI have shown significant promise as a specific RT and/or TMZ-sensitizing strategy. Ever since the rucaparib/TMZ combination was found safe to administer in patients with solid tumors (53), several studies have been launched to assess the safety and efficacy of various PARPI in patients with GBM (Table 1). The majority of early clinical trials involved patients with recurrent GBM. However, recently launched trials have involved not only newly diagnosed patients, but have also stratified patients by *MGMT* promoter methylation status to enrich the patient population likely to benefit from the therapy (NCT02152982, PARADIGM-2, and NCT03150862). Phase I or phase I/II studies in patients with recurrent GBM are helpful in determining MTD and toxicity. For example, phase I trial NCT00770471 showed that combining veliparib with RT/TMZ is not adequately tolerated (54), and based on this data, later studies planned to evaluate veliparib in combination with RT alone and/or velipaib combined with adjuvant TMZ (NCT03581292, NCT02152982), thus avoiding toxicities reported with the triple combination. Although triple combination of veliparib has been excluded from further development, other PARPI agents in combination with RT/TMZ continue to be tested (NCT03212742, NCT03150862 and PARADIGM-2).

**Table 1:** Clinical trials of various PARP inhibitors in patients with low grade gliomas and GBM.

**Clinical trial identifier**	**Sponsoring Agency**	**Description**	**Biomarker(s) as eligibility criteria**
**PHASE I STUDIES**			
NCT01390571 (OPARATIC)	Cancer Research UK	Olaparib and Temozolomide in Treating Patients with Relapsed Glioblastoma. https://clinicaltrials.gov/ct2/show/NCT01390571	None
NCT01294735	Merck Sharp & Dohme Corp.	Study of the Safety and Efficacy of MK-4827 Given with Temozolomide in Participants with Advanced Cancer (MK-4827-014 AM1). https://clinicaltrials.gov/ct2/show/NCT01294735	None
NCT00770471 (NABTT0801)	Sidney Kimmel Comprehensive Cancer Center, Johns Hopkins	ABT-888, Radiation Therapy, and Temozolomide in Treating Patients with Newly Diagnosed Glioblastoma Multiforme. https://clinicaltrials.gov/ct2/show/NCT00770471	None
PARADIGM-2	University of Glasgow	OlaPArib and RADIotherapy or olaparib and radiotherapy plus temozolomide in newly-diagnosed Glioblastoma stratified by MGMT status: 2 parallel phase I studies http://www.crukctuglasgow.org/eng.php?pid=paradigm_2	MGMT hyper-methylation to establish olaparib MTD in combination with radiotherapy and temozolomide. MGMT unmethylated - to establish olaparib MTD in combination with radiotherapy.
**PHASE I/II STUDIES**			
NCT03212742	Center Francois Baclesse, France	Study of Concomitant Radiotherapy with Olaparib and Temozolomide in Unresectable High-Grade Gliomas Patients (OLA-TMZ-RTE-01). https://clinicaltrials.gov/ct2/show/NCT03212742	None
NCT01026493 (RTOG0929)	Radiation Therapy Oncology Group	A Randomized Phase I/II Study of ABT-888 in Combination with Temozolomide in Recurrent (Temozolomide Resistant) Glioblastoma. https://clinicaltrials.gov/ct2/show/NCT01026493	None
NCT01514201	NCI	Veliparib, Radiation Therapy, and Temozolomide in Treating Younger Patients with Newly Diagnosed Diffuse Pontine Gliomas. https://clinicaltrials.gov/ct2/show/NCT01514201	None
NCT03150862	BeiGene USA, Inc.	Study to Assess the Safety, Tolerability and Efficacy of BGB-290 in Combination with Radiation Therapy (RT) and/or Temozolomide (TMZ) in Subjects with First-line or Recurrent /Refractory Glioblastoma. https://clinicaltrials.gov/ct2/show/NCT03150862	MGMT promoter methylation status (unmethylated vs. methylated)
NCT02116777	Talazoparib	Talazoparib and Temozolomide in Treating Younger Patients with Refractory or Recurrent Malignancies. https://clinicaltrials.gov/ct2/show/NCT02116777	None
**PHASE II STUDIES**			
NCT03212274	NCI	Study of the PARP inhibitor olaparib in IDH1 and IDH2 Mutant Advanced solid tumors. https://clinicaltrials.gov/ct2/show/NCT03212274	IDH1/IDH2 mutations
NCT02974621	NCI	Cediranib Maleate and Olaparib Compared to Bevacizumab in Treating Patients with Recurrent Glioblastoma. https://clinicaltrials.gov/ct2/show/NCT02974621	None
NCT03233204	NCI	Olaparib in Treating Patients with Relapsed or Refractory Advanced Solid Tumors, Non-Hodgkin Lymphoma, or Histiocytic Disorders with Defects in DNA Damage Repair Genes (A Pediatric MATCH Treatment Trial). https://clinicaltrials.gov/ct2/show/NCT03233204	Molecular Analysis for Therapy Choice (MATCH) to APEC1621H based on the presence of an actionable mutations
NCT03581292	NCI	Veliparib, Radiation Therapy, and Temozolomide in Treating Participants with Newly Diagnosed Malignant Glioma without H3 K27M or BRAFV600E Mutations. https://clinicaltrials.gov/ct2/show/NCT03581292	wild-type for H3K27M, BRAFV600E, and IDH1/2
**PHASE II/III STUDIES**			
NCT02152982 (A071102)	NCI	Temozolomide with or without Veliparib in Treating Patients with Newly Diagnosed Glioblastoma Multiforme. https://clinicaltrials.gov/ct2/show/NCT02152982	MGMT promoter hypermethylation

Another important phase I trial has been OPARATIC (NCT01390571), demonstrating that olaparib reaches tumor core and the margins in patients with recurrent GBM, and that the olaparib combined with low dose extended TMZ is well tolerated (50). This data has generated enthusiasm for the olaparib combinations in GBM. A second phase I trial (PARADIGM-2) stratifies newly diagnosed GBM based on *MGMT* hypermethylation to receive olaparib/TMZ/radiation (*MGMT* methylated) or olaparib/radiation (*MGMT* unmethylated) (55). Besides these clinical trials evaluating olaparib combinations, phase II studies NCT03233204 and NCT03212274 aim to investigate single-agent activity of olaparib in pediatric patients with mutated or altered DNA damage repair genes (NCT03233204) or in patients with IDH1/2-mutant tumors (NCT03212274). A phase-II study plans to compare the antitumor activity of olaparib combined with cediranib, an inhibitor of VEGF receptor, vs. bevacizumab monotherapy in patients with recurrent GBM (NCT02974621). An ongoing phase I-II study is investigating PARPI talazoparib combined with TMZ (NCT02116777). Children with refractory or recurrent solid tumors on this trial will receive talazoparib orally either once or twice daily on days 1–6 and TMZ on days 2–6, with therapy repeating every 28 days for up to 24 cycles until disease progression or unacceptable toxicity occurs. Due to limited distribution into the CNS in preclinical mouse models for several of these PARPI agents, concerns remain about the effectiveness of these therapies in gliomas that all have at least a partially intact BBB (56).

### Delineation of Predictive Biomarkers to PARPI-Mediated Sensitization

HR deficiency (also known as *BRCAness)* and *PARP* expression are predictive biomarkers for PARPI efficacy (57–59). However, unlike breast and ovarian cancers, *BRCAness* is uncommon in GBM. Although homozygous PTEN deletion, mutant STAG2, or IDH-mutations found in GBM have been reported to disrupt the HR pathway, these studies were performed in established cell lines (60–62). In a PDX preclinical trial, PTEN alterations had no correlation with the TMZ-sensitizing effects of veliparib (15). Similarly, veliparib had neither single agent activity nor any significant sensitization in two different IDH1-mutant GBM PDX models (data not shown). These results suggest that HR deficiency, a conventional marker of PARPI sensitivity, may not be a robust biomarker for veliparib-mediated *in vivo* sensitization in GBM.

As reported previously, PARPI-mediated *in vivo* sensitization is associated with inherent TMZ sensitivity (4, 14), whereas *MGMT* hypermethylation is a marker of TMZ sensitivity (5, 63). We assessed the utility of *MGMT* methylation status as a biomarker of veliparib-mediated sensitization in a PDX preclinical trial involving 28 GBM PDX models (15). In this preclinical trial, PDX lines with unmethylated *MGMT* had no survival benefit with TMZ/veliparib over TMZ alone, while profound survival extension with TMZ/veliparib was observed in ~45% of PDX lines with *MGMT* promoter hyper-methylation (15). Based on this result, the A071102 clinical trial uses *MGMT* promoter methylation as selection criterion for a randomized clinical trial of adjuvant TMZ combined with veliparib or placebo (NCT02152982). *MGMT* promoter methylation status has been integrated in clinical trial designs for at least two other studies testing TMZ/PARPI in GBM (PARADIGM-2, NCT03150862). However, as only a fraction of patients with *MGMT* hyper-methylation expected to benefit from TMZ/PARPI therapy, refinement of predictive biomarkers is necessary to guide optimal use of PARPI in GBM.

Lack of Schlafen Family Member 11 (SLFN11) is known to confer resistance to DNA damaging agents (64, 65). Mechanistically, SLFN11 interacts with replication protein A (RPA), destabilizes RPA-ssDNA complexes and inhibits HR (66). Like *MGMT, SLFN11* expression is epigenetically suppressed through promoter hypermethylation in nearly 50% of solid tumors (67). In a recent study, *SLFN11* expression correlated with *in vivo* tumor response to talazoparib in patient-derived xenograft (PDX) models of small cell lung cancer (SCLC) (68). Interestingly, in this study, response to TMZ/Talazoparib had no clear association with SLFN11. However, a phase II clinical trial testing TMZ plus veliparib (or placebo) in patients with SCLC showed that SLFN11-positive tumors, as defined by immunohistochemistry (*n* = 12) had improved progression-free and overall survival relative to patients with SLFN11-negative tumors (69). Based on this promising data in SCLC, SLFN11 is a potential biomarker to be examined in GBM.

IDH1 mutations are oncogenic mutations found in 74% of low-grade gliomas and 9% of GBM (70). Mechanistically, 2-HG produced by the neomorphic mutant-IDH1 enzyme inhibits α-ketoglutarate (αKG)-dependent ALKBH2-3 enzymes and prevents repair of endogenous DNA damage, rendering vulnerability to alkylation therapies (71). A recent study by Salkowski et al. suggests that 2-HG can disrupt HR activity and sensitize cells to PARPI (62). This finding was further confirmed in GBM cell lines modified to express mutant IDH1 constructs (72). However, exogenously expressed mutant IDH1 may not recapitulate all the genetic and phenotypic changes that occur in IDH1-mutant gliomas. NAD+ deficiency is one of the striking features of IDH1-mutant glioma cells, which are highly vulnerable to NAD+ depletion via TMZ treatment or NAMPT inhibition (73). Since NAD+ is consumed by PARP activation during genotoxic therapy, PARPI can be counterproductive. This hypothesis was proven by Tateishi et al. whereby TMZ/olaparib had lesser cytotoxicity than TMZ alone in glioma cells *in vitro* (74, 75). Comprehensive analysis of metabolic vulnerability is necessary to understand conflicting results of PARPI sensitivity in IDH1 mutant gliomas.

## Conclusions and Future Directions

Increased DNA repair compromises therapeutic efficacy of anti-cancer genotoxic therapies (76). Based on the pleotropic role of PARP in DNA repair, there is immense interest in clinical development of PARPI as cancer monotherapy (for HR defective tumors) and as a chemo-radiation sensitizer (76). Preclinical studies using orthotopic GBM models suggest that the efficacy of PARPI in GBM may be limited due to restricted delivery across the BBB and heterogeneous tumor response (4, 12, 13, 15). Pronounced TMZ sensitization by the brain penetrant PARPI veliparib was observed in a subset of tumors inherently sensitive to TMZ, while TMZ-resistant tumors lacked *in vivo* sensitization, suggesting that potentiation of replication stress rather than BER inhibition or PARP trapping is a key mechanism involved in *in vivo* sensitization (4, 14, 15). Based on these findings, *MGMT* promoter methylation was delineated as a predictive biomarker and is being increasingly used in PARPI clinical trials in GBM. However, as only a fraction of *MGMT* methylated tumors responded in preclinical trial, discovery of precise biomarkers is necessary.

One particular area of interest is to dissect the role of PARP in replication stress resolution. Whereas TMZ induces replicative stress via repetitive MMR at O6MeG:T sites, PARPI may potentially compromise stability of stalled replication forks (5, 7, 77). However, compromised fork protection is a complex biological process, where PARPI may act more robustly in context of vulnerabilities such as loss of BRCA1 or other factors involved in fork protection. Identification of critical regulators of fork protection in context the of TMZ/PARPI combinations will help identify new biomarkers. Endogenous replicative stress in cells with compromised fork protection may result in genomic instability and higher mutation burden. Analysis of mutation burden in the context of TMZ/PARPI therapy can be another crucial marker of PARPI-mediated sensitization. Ongoing PARPI trials are poised to generate data and biospecimens that will allow correlative analysis of putative biomarkers identified through preclinical studies in GBM models.

## Author Contributions

SG and JS conception and design. SG, ES, AM, ST, and PD acquisition of data. SG, JS, ST, and GK analysis and interpretation of data. SG, JS, SK, and GK writing, review, and/or revision of the manuscript. SG and JS study supervision.

### Conflict of Interest Statement

The authors declare that the research was conducted in the absence of any commercial or financial relationships that could be construed as a potential conflict of interest.
