# Supplementary material for: PARP Inhibitors for Sensitization of Alkylation Chemotherapy in Glioblastoma: Impact of Blood-Brain Barrier and Molecular Heterogeneity
Source: Front Oncol. 2019 Jan 22;8:670. doi: 10.3389/fonc.2018.00670 (PMC6349736; doi:10.3389/fonc.2018.00670)
Supplement: Supplementary file 1 [file Presentation_1.pdf]

Supplementary Material

PARP inhibitors for sensitization of Alkylation chemotherapy in Glioblastoma: impact of blood-brain barrier and molecular heterogeneity

Shiv K. Gupta, Emily J. Smith, Ann C. Mladek, Shulan Tian, Paul A. Decker, Sani H. Kizilbash, Gaspar J. Kitange and Jann N. Sarkaria

Methods:

Cell line, drugs & antibodies: The U251TMZ model was reported previously (Nadkarni et al, 2012). U251TMZ glioma cell line was maintained in DMEM supplemented with 10% fetal bovine serum (FBS). CyQuant assays were performed as described previously (Kitange et al, 2012). TMZ was purchased from Sigma-Aldrich (St. Louis, USA) and veliparib obtained from the Cancer Therapy Evaluation Program at NCI. Antibodies for BRCA1, BRCA2, RAD51, MPG, XRCC1, Vinculin and β-Actin were obtained from Cell Signaling Technologies.

siRNA & transfection: Silencer Negative Control #1 (cat# 4390843) and siRNA targeted against human BRCA1 (cat# 4390824), BRCA2 (cat# AM16708A, ID 121226), RAD51 (cat# 4392420), PRKDC (cat# AS0271BP, ID 103604), MPG (cat#AS021942, ID s8930) and XRCC1 (cat# AS021942, ID s14942) from Fisher-Ambion were used. For transfection U251TMZ cells were grown to 60-80% confluency in DMEM (10% FBS) medium without antibiotics in six-well plates. Cells were transfected with control or specific siRNA to 100 nM in Opti-MEM (Gibco BRL)] using Lipofectamine 2000 (Invitrogen) according to the manufacturer's instructions and were kept for 72 hours in growth medium before any further experiments.

HR assays using DR-GFP plasmid: U251TMZ cells were stably transfected with pDRGFP (Plasmid 26475, Addgene, USA) and stable Puromycin-resistant cells were selected with 5 µg/mL puromycin (InvivoGen., USA). For homologous recombination (HR) assay, U251TMZ-DRGFP cells were co-transfected with pCBASceI **(**Plasmid 26477, Addgene) with specified siRNA. Events of GFP expression by HR mediated repair of I-SceI cleaved DRGFP sites were measured by flow cytometry and presented as percent relative the control. GFP flow cytometry

was performed on live cells by BD FACS caliber and flow data analyzed using CellQuest Software.

Western blotting: Cells were processed for protein extraction and subsequent SDS-poly acrylamide gel electrophoresis as previously described (Gupta et al, 2014). Proteins (10 µg) were denatured and resolved either on 3-8% Tris-acetate gels (for high molecular weight proteins BRCA1, BRCA2, DNA-PKsc and viculin) or on 4-12% Bis-acrylamide gradient gels (for low molecular weight proteins Rad51, XRCC1, MPG and b-Actin), transferred on to PVDF membrane, blocked in 5% nonfat dry milk and then probed with appropriate primary antibodies diluted in 5% BSA (as listed below) overnight at 4ºC and followed by horseradish peroxidase-conjugated secondary antibodies. Signals were detected by using enhanced chemiluminescence method, and images captured in c300 imager (Azure Biosystems, Dublin, CA).

Primary Antibody	Source and cat #	dilution
BRCA1 (rabbit polyclonal)	Cell Signaling, cat #9010	1:1000
BRCA2 (mouse monoclonal)	Abcam, cat# ab123491	1:1000
DNA-PKcs (mouse monoclonal)	Cell Signaling, cat #12311	1:1000
RAD51 (rabbit polyclonal)	Santa Cruz, cat# sc-398587	1:1000
MPG (rabbit polyclonal)	Santa Cruz, cat# sc-101237	1:1000
Vinculin (rabbit polyclonal)	Cell Signaling # 13901	1:1000
XRCC1 (rabbit polyclonal)	Cell Signaling cat# 2735	1:5000

RNA Preparation, Sequencing and Data Analysis: Total RNA (50 ng) was extracted with the RNeasy mini kit (QIAGEN) according to manufacturer instructions. The library was sequenced using Illumina's TruSeq RNA Sample Prep Kit V2 from both ends to 101 bases on a HiSeq2000 platform. The sequencing reads from mouse were filtered out using xenome (v1.0.1, Conway et al, PMID: 22689758) from downstream analysis. Reads that were mapped to human-only and both categories were kept for downstream analysis. The filtered reads were analyzed using MAP-RSeq (v.1.2.1, Kalari et al, 2014), the Mayo Bioinformatics Core pipeline. MAP-RSeq consists of alignment with TopHat 2.0.6 (Kim et al, 2013) against the hg19 genome build and gene counts with the HTSeq (v0.5.3p9, Anders, et al, PMID: PMID: 25260700). Expression for each

gene was normalized by correcting for gene length and reported as RPKM (Reads Per Kilobase per Million mapped reads) values.

Whole-exome Seq & data analysis: Whole exome sequencing (WES) was performed in the Mayo Clinic Medical Genome Facility. Paired-end libraries were prepared and sequenced by following the manufacturer's protocol with SureSelect Human All Exon V5+UTRs (or V4+UTRs) kit from Agilent Technologies (Santa Clara, CA) on the Illumina Hiseq 2500 platform (Illumina Inc., San Diego, CA, USA).

The WES reads were aligned to the Human Reference Genome Build 37 using Novoalign (version 3.02.04) with the following options: -x 5 -i PE 425, 80 -r Random --hdrhd off -v 120 (http://www.novocraft.com/). Realignment and recalibration were then performed using GATK (version 3.3.0) by following the recommended Best Practices version (DePristo et al, PMID: 21478889). Variant calling was performed with GATK's HaplotypeCaller. Raw variant sites were subject to a series of quality filtering, such as the allelic and overall depth of coverage, average mapping quality, base quality and proximity to *homopolymer* run, number of mapping-quality-zero reads, variant quality, strand bias, and somatic score. Common variants were eliminated based on the minor allele frequencies (>0.01) available in the 1000 Genomes Project or Exome Aggregation Consortium (EXAC). Finally, tumor mutation burden was determined by the number of non-silent protein-coding mutations normalized by the captured genomic regions (per Mb).

Statistics: *In vitro* data presented are the mean ± standard error from three or more experiments. The two-sample t-test was used to make comparisons across groups. Cumulative survival distributions were estimated using the Kaplan-Meier method. The log rank test was used to make comparisons across groups.

Supplementary References

Conway T, Wazny J, Bromage A, et al. Xenome--a tool for classifying reads from xenograft samples. *Bioinformatics*. 2012;28(12):i172-8.

DePristo MA, Banks E, Poplin R, et al. A framework for variation discovery and genotyping using next-generation DNA sequencing data. *Nat Genet*. 2011;43(5):491-8.

Nadkarni A, Shrivastav M, Mladek AC, Schwingler PM, Grogan PT, Chen J, et al. ATM inhibitor KU-55933 increases the TMZ responsiveness of only inherently TMZ-sensitive GBM cells. J Neurooncol 2012;110:349–57.

Kitange GJ, Mladek AC, Carlson BL, Schroeder MA, Pokorny JL, Cen L, et al. Inhibition of histone deacetylation potentiates the evolution of acquired temozolomide resistance linked to MGMT upregulation in glioblastoma xenografts. Clin Cancer Res 2012;18:4070–9.

Gupta, S.K., Carlson, B.L., Boakye-Agyeman, F., Bakken, K.K., Kizilbash, S.H., Schroeder, M.A., Reid, J, Sarkaria, J.N. (2014) Discordant in vitro and in vivo chemopotentiating effects of the PARP inhibitor veliparib in temozolomide-sensitive versus -resistant glioblastoma multiforme xenografts. Clin Cancer Res 20, 3730-3741

Kalari, K. R., Nair, A. A., Bhavsar, J. D., O'Brien, D. R., Davila, J. I., Bockol, M. A., Nie, J., Tang, X., Baheti, S., Doughty, J. B., et al. (2014). MAP-RSeq: Mayo Analysis Pipeline for RNA sequencing. BMC Bioinformatics 15, 224.

Kim, D., Pertea, G., Trapnell, C., Pimentel, H., Kelley, R., and Salzberg, S. L. (2013). TopHat2: accurate alignment of transcriptomes in the presence of insertions, deletions and gene fusions. Genome Biol 14, R36.

Christoforides A, Carpten JD, Weiss GJ, Demeure MJ, Von Hoff DD, Craig DW. Identification of somatic mutations in cancer through Bayesian-based analysis of sequenced genome pairs. BMC Genomics. 2013;14:302. Epub 2013/05/07. doi: 10.1186/1471-2164-14-302. PubMed PMID: 23642077; PMCID: PMC3751438.

Supplementary Figure S1

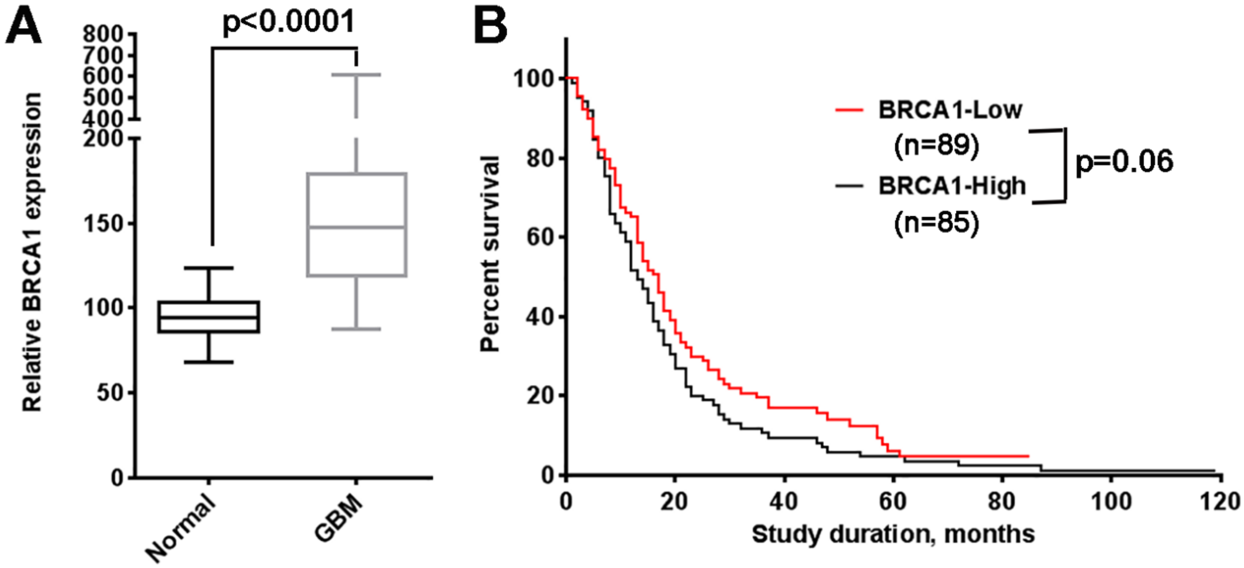

Analysis of potential prognostic significance of BRCA1 mRNA in GBM: A) BRCA1 mRNA expression in GBM tumor specimens (n=214) versus normal human brain tissue controls (n=21) reported in Rembrandt GBM online data set. **B)** Kaplan-Meier curve showing relative overall survival among GBM patients divided equally in to groups with low (n=89) versus high BRCA1 (n=89) mRNA expression.
